# Smartphone-Based Interventions and Internalizing Disorders in Youth: Systematic Review and Meta-analysis

**DOI:** 10.2196/16490

**Published:** 2021-01-11

**Authors:** Adrian Buttazzoni, Keshbir Brar, Leia Minaker

**Affiliations:** 1 School of Planning University of Waterloo Waterloo, ON Canada; 2 School of Public Health and Health Systems University of Waterloo Waterloo, ON Canada

**Keywords:** mental health, meta-analysis, mobile phone, smartphone, systematic review, youth

## Abstract

**Background:**

Mental health disorders in youth are a global issue that have important implications for the future quality of life and morbidity of affected individuals. In the context of public health initiatives, smartphone-based interventions have been suggested to hold the potential to be an effective strategy to reduce the symptoms of mental health disorders in youth; however, further evaluation is needed to confirm their effectiveness. This systematic review and meta-analysis documents and synthesizes existing research on smartphone-based interventions targeting internalizing disorders in youth populations.

**Objective:**

This study aims to synthesize existing research on smartphone-based interventions targeting internalizing disorders in youth populations.

**Methods:**

PubMed and SCOPUS were searched in 2019, and 4334 potentially relevant articles were found. A total of 12 studies were included in the final synthesis. We used the Hedges *g* meta-analysis approach and a random effects model for analysis.

**Results:**

The results of this review note that depression and anxiety are the most commonly targeted symptoms, and unlike other similar topics, most studies reviewed were linked to a proven treatment. The overall pooled effect from the meta-analysis showed small but significant effects (κ=12; N=1370; Hedges g=0.20; 95% CI 0.02-0.38) for interventions in reducing the symptoms of internalizing disorders. In total, 4 subgroup analyses examining specific symptoms and intervention styles found varied small significant and nonsignificant effects.

**Conclusions:**

Future research should focus on developing robust evaluative frameworks and examining interventions among more diverse populations and settings. More robust research is needed before smartphone-based interventions are scaled up and used at the population level to address youth internalizing disorders.

## Introduction

Globally, between 10% and 20% of children and adolescents experience mental illness, with approximately half of all mental illnesses known to begin by the age of 14 years [1]. Poor mental health during these developmental years has been linked with unfavorable outcomes regarding employment, relationships, family formation, and health and disability in early adulthood [2]. Childhood and adolescence are key life stages for interventions with regard to mental health and well-being. However, at present, the screening and support for youth mental health issues in many primary health care systems continue to be inadequate, as even optimistic estimates suggest that only 50% of illnesses are detected by doctors [3]. New and innovative strategies are needed to help address mental health issues in youth. Smartphone-based interventions have been increasingly employed to track symptoms and provide support for individuals on a range of related health issues, such as concussions [4]. Taking this potential and the relatively high digital and tech literacy of younger populations into consideration, there has been a recent growth in the interest of delivering mental health interventions to youth cohorts via smartphones [5].

Internalizing disorders are those in which individuals tend to express distress inwardly, such as anxiety disorders and mood disorders (eg, major depressive disorder [6]). Anxiety is one of the most common disorders in young people and is estimated to affect 4% to 20% of children and adolescents [7], whereas depression is said to affect 2% to 8% of children and adolescents [8] and is a leading cause of disability globally [9]. Similarly, stress is often internalized and can significantly impact an individual’s coping abilities, self-esteem, and social relations [10], whereas insomnia (ie, sleep problems) has a robust relationship with depression [11] and other internalizing symptoms including anxiety [12]. Disorders can begin early in childhood and can develop into chronic conditions that negatively impact an individual’s relationships, development, and daily functioning in the near future [13]. These disorders are associated with functional impairment, increased risk of depression and suicide [14], and substance abuse issues [15] in the long term. As a result, internalizing disorders carry the potential for high societal burdens [16].

Recently, smartphones have become an essential tool in the targeted support, management, and monitoring of mental health disorders. Common mental health intervention strategies using smartphones include text messaging services [17] and smartphone apps [18]. Growing evidence supports the feasibility and potential of smartphone-based interventions to address mental health issues and disorders [19]. For example, positive effects have been observed regarding memory training for older adults [20] and for people with attention-deficit or hyperactivity disorder [21]. Consequently, smartphones are being increasingly used to address mental health issues of youth and adolescent populations [22]. However, with an increase in the use of smartphones as an intervention delivery strategy, there is a need to improve the evaluation aspect of such interventions [23].

### Justification for Review and Meta-Analysis

A recent systematic review of meta-analyses that focused on internet- and mobile phone–based interventions for mental and somatic conditions among children found 8 relevant meta-analyses [24]. Of the included papers, 5 focused on web-based or computerized interventions, one was primarily concerned with psychological-based interventions and one searched for but did not include phone-based interventions. The single meta-analysis that included smartphone-based interventions only analyzed one mobile phone–based intervention study [25]. Another recent meta-review [22] broadly cataloged and synthesized existing reviews of all types focusing on digital health interventions for young people with mental health problems. This review did not report any other meta-analyses (although 2 additional relevant scoping reviews and 1 additional systematic review were reported) beyond those documented by Domhardt et al [24]. To date, no meta-analysis has been conducted that has sought to quantitatively, and solely, evaluate the effectiveness of smartphone apps with regard to mental health (specifically, internalizing disorders) of youth populations. This review and meta-analysis are the first to aim to address these points and present an evaluation.

### Review Question and Objective

The primary research question guiding this review and meta-analysis is as follows: What are the study designs, intervention features (review), and effectiveness (meta-analysis) of smartphone-based interventions that aim to minimize or reduce the symptoms of youth internalizing disorders? By applying the population, intervention, comparison, outcome, and context model of Petticrew and Roberts [26], we operationalized our meta-analysis research question as presented in Textbox 1.

**Population, intervention, comparison, outcome, and context review research question breakdown (criteria and description).**

- Population: youth (in general, <18 years old; however, in some articles, youth was defined as ≤24 years)
- Intervention: smartphone-based interventions specifically targeting an internalizing disorder in a youth population
- Comparison: control versus intervention, group 1 versus group 2, time 1 versus time 2, etc
- Outcome of interest: effectiveness of intervention in reducing symptoms or intensity of internalizing disorder (ie, anxiety, depression, insomnia, stress)
- Context: any

Following this question, the principal objective of the meta-analysis is to quantitatively evaluate the effectiveness of smartphone-based interventions with regard to youth internalizing disorders.

## Methods

### Search Strategy

Searches of electronic databases were used to identify and document the articles presented in this review and the meta-analysis in June 2019. Different variations of, and other common terms used for, each focal concept (ie, smartphone, internalizing disorder, and youth population) were discussed and developed by the authors, whereas specific terms were truncated as necessary (Textbox 2).

**Search strategy outline.**

- Smartphone

“cell phone” OR “cellular phone” OR “mHealth” OR “mobile health” OR “mobile phone” OR “phone” OR “SMS” OR “short message service” OR “smartphone” OR “text” AND

- Internalizing disorder

“anxiety” OR “depression” OR “internalizing disorder*” OR “internalising disorder*” OR “insomnia” OR “stress” AND

- Youth population

“adolescent*” OR “child*” OR “teen*” OR “youth” OR “young adult”

When deciding on which electronic databases to select for this review, we needed to ensure that content from the fields of behavioral science, pediatrics, psychology, and public health are captured. Therefore, to best incorporate this diversity in content and research areas, we conducted the search strategy in 2 interdisciplinary databases—PubMed and SCOPUS.

### Eligibility Criteria

A total of 5 specific criteria were applied to the article search. The criteria stipulated that each article must (1) be focused primarily on the implementation and evaluation of a smartphone-based intervention (ie, not web-based strategies, social media focused initiatives, etc); (2) have reported some description of the study design and sample, as well as the intervention strategy, implementation, evaluation, and targeted outcome of the intervention strategy; (3) have been a primary research study (ie, not a review, feasibility or acceptability study, proposal, technical report etc) in the design of a randomized controlled trial (RCT), case control, cohort, or cohort analytic design; (4) have focused on and reported the outcome for one or more internalizing disorder outcomes (ie, anxiety, depression, insomnia, stress); and (5) be written in English. There were no geographic or publishing date restrictions placed on the search.

### Study Selection and Review Process

**Figure 1:**
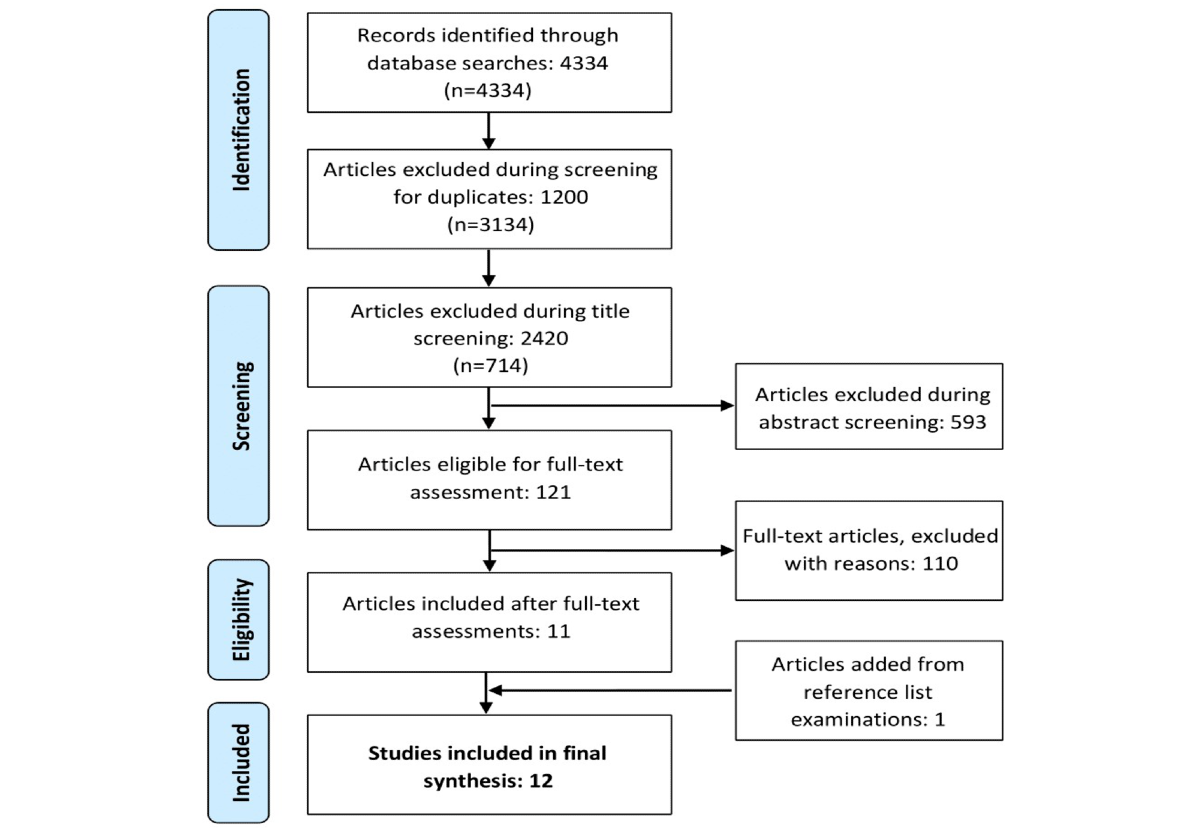
Flow diagram of study selection processes.

Initial searches of PubMed (n=1726) and SCOPUS (n=2608) returned a total of 4334 results (Figure 1). Removal of duplicates resulted in 1200 titles being discarded. The remaining 3134 titles were vetted next, after which 2420 articles were excluded. Abstracts were then screened, resulting in 593 more records being removed. After full-text assessments of the final 121 articles were completed, a total of 11 papers were deemed to meet all of the eligibility criteria. Having included studies that primarily examined adolescents but were mixed with young adult populations, we decided to include 3 studies [27-29] with similar sample demographics but had a mean sample age >18 years and did not explicitly refer to their participants as *adolescents* or *youth*. The most common reasons for exclusion in the full-text assessment phase were feasibility studies lacking a formal assessment, proposal articles only presenting concepts, research papers not including a youth population, and primary studies not reporting an internalizing disorder outcome. Of the total, 1 paper [30] met all of the above conditions; however, its reporting of the outcome of interest was not precise enough to be included in the analysis. Reference list searches of the included articles added 1 additional article to the review. Eventually, 12 studies were documented and synthesized in the systematic review and subsequently included in the meta-analysis. Initial title and abstract vetting was conducted by a team member (AB) with a second member (LM) spot checking the abstract vetting to ensure the consistency of the process. Similar protocols were carried out for the later full-text assessment and data extraction phases (AB and LM).

### Systematic Review Data Extraction

Systematic review data extractions and meta-analysis coding data for all the included articles are available in the Multimedia Appendix 1. The following information was gathered from each study: citation information, study design information (eg, primary outcome evaluated), smartphone-based intervention details (eg, supporting resources, delivery methods, length), sample details, and internalizing disorder variable information (eg, outcome measure used).

### Variables and Concepts Included in Meta-Analysis

*Internalizing disorders* are often defined as any depressive and anxiety condition [31]. However, other definitions of internalizing disorders have expanded the concept to include panic and stress [32,33], whereas insomnia has become an important symptom of depressive and anxiety disorders [31]. Therefore, this meta-analysis conceptualized internalizing disorders as *anxiety*, *depression*, *insomnia*, or *stress*. *Smartphone-based interventions* were defined as any app, initiative, program, promotion, service, or tool that is based or run primarily through a smartphone.

### Meta-Analysis Coding

In addition to the data extractions for the systematic review portion of this manuscript, identified studies pooled in the meta-analysis were also coded to include statistical data. On the basis of the availability of reported results from each study, statistics coded specifically for the meta-analysis included intervention effect sizes (eg, available between- or within-group *d* values and their corresponding SDs), results of *t* tests (eg, differences in means) in cases where no *d* values were reported, scale reliability scores, scale anchors, data source report (eg, self, other), and, if applicable, any information pertaining to multiple measures being employed in the same study.

### Meta-Analysis Approach and Statistical Procedures

To guide the meta-analysis, we drew from the outline for effect size meta-analyses of Hunter and Schmidt [34] and chose to use the Hedges g approach. In traditional *d* value meta-analysis approaches, such as the *bare bones* method, no corrections are made for any artifacts other than sampling error [34]; however, given some of the small sample sizes of the included studies, we elected to follow previous recommendations [35] which stipulate using Hedges g as a correction for effect size—a method that pools weighted** **SDs unlike Cohen *d*. Similar to others [36], we elected to use a more conservative approach given the relatively small number of primary articles being used to estimate the pooled intervention effects. The reported or calculated effect size for the interventions was used to assess the between-group or within-group effectiveness of the interventions on reducing or minimizing internalizing disorder symptoms. Importantly, among the 8 between-group studies, 5 were randomized. Once the *d* values from each study (*uncorrected effect sizes*) were derived, we applied the Hedges g formula to calculate *corrected effect sizes* [35]. A random effects model was used for this meta-analysis because, unlike fixed effects models, it allows for potential variation in underlying parameters. As such, random effects models have been argued to more accurately reflect the real-world heterogeneity of effects [37].

As a part of our conservative approach, all intervention follow-up values used in the meta-analysis were the last date (eg, 2-week and 4-week follow-ups; 4-week selected) reported in each study. Statistics calculated in the meta-analysis included the mean unweighted observed effect, mean effect size weighted by sample size, SD of the sample-weighted effect, corrected weighted variance of the effect (ie, Hedges g), and percentage of variance because of sampling error. We also calculated the same statistics for 4 separate subgroups (specific symptoms and intervention style—2 subgroups each) of the studies. In cases where multiple outcomes of interest were reported in a single study, we calculated the average standardized difference across variables to ascertain the overall effect size [34]. If no effect size data were provided, we followed the method by Morris and DeShon [38] of pooling pre- and posttest means and dividing them by the pretest SD to calculate effects. Sensitivity analyses following the *Cochrane Handbook for Systematic Reviews of Interventions* method [39] were used to calculate effect sizes for articles that only reported baseline and follow-up means (ie, no changes in means data) and to subsequently impute an SD of the change for the experimental group. All analyses were completed in Microsoft Excel.

### Quality and Risk of Bias Assessments

Quality assessments (QAs) of the 12 studies reviewed and analyzed are shown in Table 1. To conduct the assessments, we used the Effective Public Health Practice Project (EPHPP) QA tool [40]. Global ratings for each study were developed following the EPHPP dictionary guidelines; 2 reviewers (AB and KB) first calculated independent scores for each article and subsequently evaluations were compared [41]. Comparisons of the evaluations were undertaken to address and resolve any grading variability and other interpretation differences between the 2 reviewers. Reviewers graded each article on its selection bias, study design, confounders, blinding, data collection, withdrawals, and dropouts. Ex-post comparisons of scores between the 2 separately completed individual assessments resulted in an interrater reliability for the QAs of over 90%. The final results of the QA examination were mixed with 4 articles possessing a strong global rating, 5 with a moderate rating, and 3 with a weak rating.

**Table 1:** Quality assessment of the articles included in meta-analysis.

Study (reference)	Selection bias	Study design	Confounders	Blinding	Data collection	Withdrawals and dropouts	Global rating^a^
Clarke et al 2016 [27]	Moderate	Moderate	Moderate	Weak	Strong	Weak	Weak
Grassi et al 2009 [28]	Moderate	Moderate	Strong	Weak	Strong	Weak	Weak
Kauer et al 2012 [42]	Moderate	Strong	Strong	Moderate	Strong	Strong	Strong
Lee et al 2013 [43]	Weak	Strong	Strong	Moderate	Strong	Weak	Weak
Ranney et al 2016 [44]	Moderate	Moderate	Strong	Weak	Strong	Strong	Moderate
Ranney et al 2018 [45]	Moderate	Strong	Strong	Moderate	Strong	Strong	Strong
Reid et al 2011 [46]	Moderate	Strong	Strong	Moderate	Strong	Moderate	Strong
Stallard et al 2018 [47]	Moderate	Moderate	Strong	Weak	Strong	Strong	Moderate
Takahashi et al 2019 [29]	Moderate	Moderate	Strong	Weak	Strong	Strong	Moderate
Werner-Seidler et al 2019 [48]	Strong	Moderate	Strong	Weak	Strong	Moderate	Moderate
Whittaker et al 2017 [49]	Moderate	Strong	Strong	Strong	Strong	Strong	Strong
Worthen-Chaudhari et al 2017 [50]	Moderate	Moderate	Moderate	Weak	Strong	Strong	Moderate

^a^Per the global rating system, strong: no weak ratings; moderate: 1 weak rating; and weak: 2 or more weak ratings.

More comprehensive risk of bias assessments was also completed for each study included in this review and meta-analysis. A total of 2 separate tools were employed in this regard: nonrandomized intervention papers’ risk of bias was assessed using the ROBINS-I (Risk Of Bias In Non-randomised Studies-of Interventions) tool for nonrandomized studies of interventions [51], whereas randomized interventions’ (ie, RCTs) risk of bias was assessed using the Cochrane Collaboration tool for assessing risk of bias in randomized trials [52]. Risk of bias assessments of the nonrandomized interventions concluded with all 7 papers being scored as at a *serious risk of bias* overall, whereas evaluations of the randomized trials resulted in 4 of 5 papers being rated as at a *low risk of bias*. Regarding the former result, this trend was largely a consequence of the rating system stipulating that one rating of *serious risk* in any category will be scored with an overall *serious risk of bias* judgment for an article [51]. More specifically, the most common source of potential bias among nonrandomized studies was related to uncertainties regarding the blinding of assessors with regard to the allocation or intervention status of participants, which is not possible in single group interventions (4 out of 7 papers in this portion of the review). Visual illustrations of the results can be found in Multimedia Appendices 2 and 3.

## Results

### General Characteristics of Included Studies

The characteristics of the 12 included studies are presented in Multimedia Appendix 1. In total, 5 studies were conducted in Australia or New Zealand, 3 in the United States, 2 in Europe, and 2 in Asia. Regarding the evaluated sample populations, 10 of the 12 studies examined between 20 and 120 participants (mean 114), 8 studies had a majority of female participants, 2 had an even split, and 2 had a majority of male participants. Depressive symptoms were the most common specific symptom addressed (in 9 studies), followed by anxiety symptoms (in 6 studies). Insomnia and stress symptoms were infrequently targeted and were only included in studies with multiple outcomes. Among the studies, 9 explicitly provided relevant details or numbers; no intervention had a retention rate lower than 70%, with 7 noting rates above 90%.

### Systematic Review

Several interventions reported multiple follow-ups during the course of their evaluation. The range of follow-up periods varied from same day postoperation [43] to 12 months [49]. A number of different scales were used across studies to measure the internalizing disorder outcomes. In fact, the only scales used multiple times were the Beck Depression Inventory-II, Depression Anxiety Stress Scale, and Center for Epidemiological Studies–Depression Child. Most studies were conducted in a *real-world* setting, such as at home or in school locations. Other contexts included emergency rooms [44,45], medical clinics [50] or hospitals [43], and commuting settings [28]. A variety of different program delivery methods (eg, apps, monitoring programs, text messages, videos) were reported among the included studies. Only 3 studies [28,43,49] did not examine populations particularly affected by or at risk of internalizing disorders (eg, *at risk* diagnosed disorder, history of self-harm, etc).

Furthermore, 8 of the 12 studies reported an empirically proven treatment or guideline on which their intervention was based. Cognitive behavioral therapy (CBT) was the most commonly reported treatment, with 5 studies noting its use. Other treatments listed were attention bias modification [27], emotional self-awareness [42], and positive psychology, social interaction, and gameful design [50]. Among the CBT-centered interventions, specific features included concepts such as emotional regulation [45], thought modification [44], photo libraries of positive memories and physical activities [47], video diary messages [49], and psychoeducation [48]. Documented intervention delivery strategies in the non-CBT studies included visual relaxation narratives [28], smartphone app games [43], stress monitoring [46], attention bias modification task completion exercises [27], daily reporting of moods, substance use, sleep, and activities [42], symptom frequency and severity tracking [50], and videos and positive messaging [29].

### Effectiveness of Interventions (Meta-Analysis)

The meta-analysis examined smartphone-based interventions as 1 total group and based on primary outcome and intervention style. Table 2 shows the results of the overall and subgroup meta-analyses. The pooled unweighted sample effect sizes of all studies (κ=12; N=1370) reflect a small-to-moderate effect (*d*=0.40). When sample weights are added to calculate a more credible estimate, a smaller but significant mean effect size (*d*=0.20; 95% CI 0.02-0.38) was observed. The sampling error variance explains approximately 3.5% of the variance in this corrected estimate. The subgroup analysis of intervention studies primarily assessing anxiety symptoms (κ=6; n=322) had a greater sample-weighted standardized effects mean compared with the overall group and was statistically significant (*d*=0.42; 95% CI 0.00-0.83). Among a larger sample, interventions targeting depressive symptoms (κ=9; n=1102) had a notably smaller effect that was similarly significant (*d*=0.16; 95% CI 0.01-0.31). When analyzing the interventions based on their predominant style and features, the sample-weighted mean effect was significant and greater for the group of all other styles (eg, monitoring, relaxation, support, not CBT) of program delivery (κ=7; n=380; *d*=0.42; 95% CI 0.09-0.75). Interventions using CBT features had a very small weighted mean effect size (κ=5; n=990; *d*=0.11; 95% CI −0.06 to 0.28) but were nonsignificant.

**Table 2:** Effectiveness of smartphone-based interventions on reducing youth internalizing disorder symptoms, overall, by primary targeted outcome, and by intervention style.

Analysis		κ^a^	N^b^	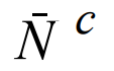	g^d^	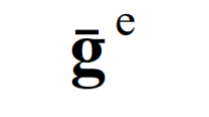	SD_wg_^f^	S_*g_^g^	Variance sampling^h^ (%)	95% CI^i^
Total		12	1370	114	0.40	0.20	0.07	0.11	0.04	0.02 to 0.38
**Symptom**										
	Anxiety^j^	6	322	54	0.43	0.42	0.24	0.32	0.08	0.00 to 0.83
	Depression^j^	9	1102	122	0.28	0.16	0.03	0.06	0.03	0.01 to 0.31
**Intervention style**										
	CBT^k^-based	5	990	198	0.19	0.11	0.03	0.05	0.02	−0.06 to 0.28
	Other (eg, monitoring, support, relaxation)	7	380	54	0.55	0.42	0.12	0.20	0.08	0.09 to 0.75

^a^κ: number of studies.

^b^N: total sample size for studies combined.

^c^*
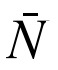
*: average sample for studies combined.

^d^g: unweighted mean Hedges g*.*

^e^*
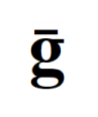
*: sample-weighted mean effect size (ie, weighted average of Hedges g).

^f^*SD*_wg_: SD of the sampled-weighted effects.

^g^S_*g_: corrected weighted variance of the effect (Hedges g).

^h^Percentage of variance sampling: percentage of variance because of sampling error (ie, average sampling error variance).

^i^95% CI: CIs at 95% for sample-weighted mean.

^j^Studies with multiple outcomes were included in the subgroup analysis of both anxiety and depression.

^k^CBT: cognitive behavioral therapy.

## Discussion

### Principal Findings

This meta-analysis reviewed the features and explored the effectiveness of smartphone-based interventions aimed at addressing youth internalizing disorders. The review findings indicated that the majority of interventions were grounded in proven treatments, that depressive and anxiety symptoms were the predominant outcomes measured and evaluated, and that all studies were conducted in developed nations. Across the 12 studies and 1370 participants included in the meta-analyses, and after being corrected for sample error variance, a small but significant pooled effect was observed with regard to reducing internalizing disorder symptoms. When examining subgroups based on specific symptoms and intervention styles, potentially because of small κ values, mixed results were observed among the 4 analyses.

Findings from this review indicate that research conducted on the effectiveness of smartphone-based interventions for youth internalizing disorders appears to be in the nascent stages but is growing. A global review assessing the overall state of mobile health apps documented 3673 mobile phone apps but found that only 247 corresponded with published papers [53]. However, although evaluative studies are still being conducted, this particular body of literature may be growing. A similar review conducted 6 years ago documented smartphone app interventions for depression among all populations and synthesized only 8 papers [54]; this review synthesized 12 studies for 1 specific demographic. Given that during our full-text assessments, we discarded over 15 potential papers for being in the pre-evaluation phase (ie, they were one of a protocol, feasibility, or acceptability manuscript), more evaluations on this topic appear likely in the coming years.

Our review also documented that unlike findings in other related health fields, the majority of studies on this topic have been linked to proven treatments. Researchers studying smoking cessation, for instance, found that less than a third of web-based interventions were linked to proven treatment or guidelines [55]. Others examining depression apps [56], for instance, have described similar trends. Similarly, an examination of the National Health Service app library in the United Kingdom revealed that only 4 of 27 apps provided any form of evidence related to patient-reported outcomes in support of their claims of effectiveness [57], which has subsequently raised concerns about the reliability of these tools as support mechanisms. This review found that 66% (8/12) studies linked their intervention to a proven treatment, which represents an encouraging development in this regard and a chance to reiterate that such research practices should be applied in all appropriate future work.

The results of the meta-analysis confirm the tendency of digital public health interventions to produce modest-to-low effect sizes. Similar trends were reported in a systematic review and meta-analysis of internet-based promotions for health behavior change by Webb et al [58], who concluded that such “interventions had a statistically small but significant effect on health-related behavior.” Typically, these outcomes are argued to be a result of a myriad of confounding variables (eg, genetic predispositions, culture, seasonality); however, the mixed effectiveness of interventions targeting youth populations has previously been considered to derive from poor or insufficient efforts to adapt the initiatives to children’s cognitive and developmental needs [59]. A lack of professional support, tailoring to individual needs, and formulation taking into account immediate family and school contexts have been specifically noted [60]. Given that tailoring or personalization of public health interventions has been found to be desirable [61], it may be worthwhile for future interventions dealing with younger populations to incorporate such strategies, especially those pertaining to cognitive abilities and app support. In fact, in a study of Australian adolescents who were enrolled in a digital intervention, participants professed to value the program’s relatability, narrative structure, and personal choice aspects [62]. Interestingly, however, across the reviewed studies in this review there were, generally, relatively high retention rates—unlike earlier reviews on similar topics such as computer-tailored intervention for behavior change (74.4%) [63]—although, this is likely because of many small-scale evaluations. Nevertheless, an enhanced emphasis on these aspects of program delivery going forward may help improve the effectiveness of smartphone-based interventions addressing youth populations.

Cross-study assessments of specific internalizing disorder symptoms (eg, anxiety) and intervention style (eg, CBT based) in our subgroup analyses revealed mixed significant effects. Interestingly, studies that included interventions featuring CBT, which is a therapeutic approach that has been recommended for the treatment of mental health issues among adolescents [64], only produced a small, nonsignificant pooled effect size. This may possibly be a consequence of such interventions still being in their nascent stages and having only been tested on limited populations. For comparison, Webb et al [58] similarly observed small effects in their overall analyses of internet-based interventions; they also found no significant effects for smaller subgroup analyses (eg, smoking abstinence, model or demonstration behavior change techniques). There are a few other possible reasons for this outcome. First, this was likely a consequence of the Whittaker et al [49] article’s relatively large sample and correspondingly small effect exerting its influence over the other smaller studies. Second, known barriers to CBT include a lack of training, infrastructure, and funding [65], which may have been present in some studies. Finally, our conservative approach, specifically the decision to use the last follow-up reports and not the most recent postintervention reports resulted in the smallest calculations of Hedges g values (effect sizes) used in the final meta-analysis.

Taking the review and meta-analysis results together, future studies are warranted to better understand the specific impacts of smartphone-based interventions on different internalizing disorder symptoms as well as their effectiveness as a public health program delivery method. Noting the suggested potential of theory-informed interventions from the review of Webb et al [58] and the small but significant effects found in this review, it would be prudent for future interventions targeting youth mental to continue including proven treatments and pair them with behavioral or social change theories in their delivery methods. A similarly important area will be developing robust evaluation frameworks. As we identified varying lengths of follow-up evaluations, a diverse range of assessment scales, and a variety of different delivery strategies (eg, text messages, apps, monitoring) among the included studies, developing methods to precisely understand and evaluate smartphone-based interventions for sustainability, efficacy or user satisfaction, and functionality should be a priority. On this point, Chan et al [66] have previously recommended that apps be evaluated based on 3 central criteria: integration or infrastructure, usability, and usefulness. Future evaluative frameworks may also consider criteria related to support in the form of self-help strategies, as the method has the potential to relieve some of the burden on existing health care services [67] and has provided positive results for mental health interventions in individual research studies [68].

### Limitations

There are important caveats to note when interpreting the results of this meta-analysis. As an area of study, research on smartphone-based interventions targeting youth and young people’s mental health, specifically internalizing disorders, is still emerging when compared with other areas of public mental health research. Many of the corrected estimates presented in this meta-analysis were thus derived from small sample populations and a limited number of studies overall. In addition, owing to these small numbers, we did not correct for any other potential errors such as attenuation or dichotomization in the meta-analysis. Such analytical limitations are important to disclose given that the effects of public health interventions are, as previously noted, typically confounded by several variables that may not be captured in the measurement of the primary outcomes and additional corrections could enhance the insights of a meta-analysis. Similar to other recent meta-analyses of health behavior change interventions [69], our risk of bias assessments varied widely across evaluated studies and their intervention designs and should be considered in the context of potential assessor biases being present. Finally, several different scales and measures were used to assess internalizing disorders, potentially resulting in some discrepancy in the measurements of the outcomes used in the analysis. Although the observed between-study variance because of sampling noted in our results was rather modest, which is likely because of the overpowering impact of the Whittaker et al [49] sample and age restrictions of this review—there is likely a high level of variation in our findings derived from the clinical and methodological heterogeneity (eg, varying levels of randomization, types of interventions, controls in analyses) of included studies. On the basis of this, we encourage more specifically focused future meta-analyses that contain a greater number of studies to assess these forms of heterogeneity in their analyses.

### Conclusions and Future Recommendations

Smartphone-based interventions targeting youth populations appear to be an efficacious strategy to address symptoms of internalizing disorders. This systematic review and meta-analysis found small but significant pooled effects sizes for smartphone-based interventions in reducing the symptoms of internalizing disorders among youth. However, the results also clarify the need for more research in this area. More empirical research studies conducted on a wider range of populations and settings and development of evaluative frameworks for smartphone-based intervention are recommended for future study. Furthermore, our meta-analysis confirms that only a few of the identified conference proceedings, feasibility studies, and other reports have been comprehensively and rigorously evaluated. By following these suggestions, it is possible to further improve not only the understanding of the impact of smartphone-based interventions on youth populations but also better assess the efficacy of smartphones as a mechanism of change for youth internalizing disorders.

## Appendix

Multimedia Appendix 1 General characteristic of the included studies.

Multimedia Appendix 2 Risk of bias in randomized studies.

Multimedia Appendix 3 Risk of bias in nonrandomized studies.

## Abbreviations

CBT: cognitive behavioral therapy
EPHPP: Effective Public Health Practice Project
QA: quality assessment
RCT: randomized controlled trial
