# Supplementary material for: Smartphone-Based Interventions and Internalizing Disorders in Youth: Systematic Review and Meta-analysis
Source: J Med Internet Res. 2021 Jan 11;23(1):e16490. doi: 10.2196/16490 (PMC7834929; doi:10.2196/16490)
Supplement: Multimedia Appendix 1 [file jmir_v23i1e16490_app1.docx]

**Table 2.** General characteristics of included studies.

| Study | Location | Sample^a^ details: tN (c/i)^b^; mean age in years (gender breakdown^c^) | Condition | Outcome measure | Study design | Intervention description, follow-up, (guideline/treatment) |
| --- | --- | --- | --- | --- | --- | --- |
| Clarke et al  (2016) [27] | Australia | 36 (c:18/i:18); 19.1 (75% female) | Insomnia-related anxiety | APSQ^d^ | Cohort analytic^e^ | Task completion, symptom tracking, and task completion on phone, 8-day intervention (attention bias modification) |
| Grassi et al (2009)^f^ [28] | Milan and Varese, Italy | 60 (i1:30/i2:30); 23 (50% female) | Anxiety | STAI^g^ | Cohort analytic^e^ | Relaxation, visual narratives on phone, 2-day intervention (N/R^h^) |
| Kauer et al (2012) [42] | Victoria, Australia | 86 (c:36/i:50) 17.4/18.5 (73% female) | Depression | DASS^i^ | RCT^j^ | Self-monitoring, mobile-type program on phone, 6-week follow-up (emotional self-awareness) |
| Lee et al (2013) [43] | Korea | 40; 5.7 (35% female) | Anxiety | mYPAS^k^ | RCT | Anxiety reduction, game apps, postoperation anxiety, immediate follow-up (N/R) |
| Ranney et al (2016) [44] | Northeast United States | 16; 15.4 (50% female) | Depression | BDI-II^l^ | Single cohort pre-post | Mood change, text messages, *iDOVE* intervention, 16-week follow-up (CBT^m^) |
| Ranney et al (2018) [45] | Northeast United States | 116 (c:58/i:58); 15.1/14.8 (58% female) | Depression | BDI-II | RCT | Goal-setting, text messages, reflection, *iDOVE* intervention, 16-week follow-up (CBT) |
| Reid et al (2011) [46] | Victoria, Australia | 114^n^ (c:46/i:68); 17.4/18.5 (69% female) | Depression, anxiety, stress | DASS (all outcomes) | RCT | Self-monitoring, mobile-type program on phone, 6-week follow-up (N/R) |
| Stallard et al (2018) [47] | Multiple locales in United Kingdom | 40; 16 (91% female) | Depression, anxiety | MFQ^o^ (depression), RCADS^p^ (anxiety) | Single cohort pre-post | Mood-lifting activities, BlueIce smartphone app, 12-week follow-up (CBT) |
| Takahashiet al (2019) [29] | Takahashi, Japan | 22; 20 (27% female) | Depression | CES-DC^q^ | Single cohort pre-post | Improving self-confidence, *SPSPS* smartphone app, motion picture-reproducing app, 5-week follow-up (N/R) |
| Werner-Seidler et al (2019) [48] | Australia | 32-34^r^; 13.7 (66% female) | Depression, insomnia, anxiety | PHQ-A^s^ (depression), ISI^t^ (insomnia), GAD-7^u^ (anxiety) | Single cohort pre-post | Education, sleep-ninja smartphone app, 6-week program (CBT) |
| Whittaker et al (2017) [49] | Auckland, New Zealand | 786 (c:392/i:394); 14.3/14.3 (74% female) | Depression | CDRS-R^v^, RADS-2^w^, MFQ | RCT | Key messaging, daily videos and text messages, 12-month follow-up (CBT) |
| Worthen-Chaudhari et al (2017) [50] | Cincinnati, United States | 22 (c:12/i:10); 15.6 (70% female) | Depression | CES-DC | Cohort analytic | Support, symptom tracking, mobile phone health app as a gamified symptoms journal, 3-week follow-up (positive psychology, social interaction, gameful design) |

^a^Samples based on participants analyzed in final follow-up, not baseline reporting.

^b^tN: total sample (c=control/i=intervention).

^c^Final sample population numbers reported and analyzed in this review and analysis may not accurately correspond with the gender breakdowns reported in the individual studies (ie, the gender reports were done in reference to initial sample and we used the final numbers analyzed after attrition and dropouts in each of the papers).

^d^APSQ: Anxiety and Preoccupation About Sleep Questionnaire.

^e^Cohort analytic design is reference to a nonrandomized two-group, pre-post analysis.

^f^The paper analyzed 4 different groups, but for the purposes of the meta-analysis, we only included the 2 intervention groups that received a mobile phone–based intervention.

^g^STAI: State-Trait Anxiety Inventory.

^h^N/R: none reported.

^i^DASS: Depression Anxiety Stress Scale.

^j^RCT: randomized controlled trial. The study was reported as an RCT; however in our analysis, we treated the design as a single-arm cohort pre-post because of multiple interventions being compared in the analysis (ie, not a traditional control vs intervention design).

^k^mYPAS: modified Yale Preoperative Anxiety Scale.

^l^BDI-II: Beck Depression Inventory-II.

^m^CBT: cognitive behavioral therapy.

^n^The paper included multiple subgroups within the intervention group, of which we used 3 of 4 (one outcome beyond the scope of analysis) in the analysis; however, because of uncertainty regarding duplicate participants among the different groups, we used the total final reported analyzed sample to be conservative.

^o^MFQ: Mood and Feelings Questionnaire.

^p^RCADS: Revised Child Anxiety and Depression Scale.

^q^CES-DC: Center for Epidemiological Studies–Depression Child.

^r^Different sample sizes corresponded with different measured outcomes.

^s^PHQ-A: Patient Health Questionnaire-Adolescent version.

^t^ISI: Insomnia Severity Index.

^u^GAD-7: Generalized Anxiety Disorder-7.

^v^CDRS-R: Children’s Depression Rating Scale-Revised.

^w^RADS-2: Reynold adolescent depression rating scale-2nd edition.
