# Supplementary figures and images for: Smartphone-Based Interventions and Internalizing Disorders in Youth: Systematic Review and Meta-analysis
Source: J Med Internet Res. 2021 Jan 11;23(1):e16490. doi: 10.2196/16490 (PMC7834929; doi:10.2196/16490)

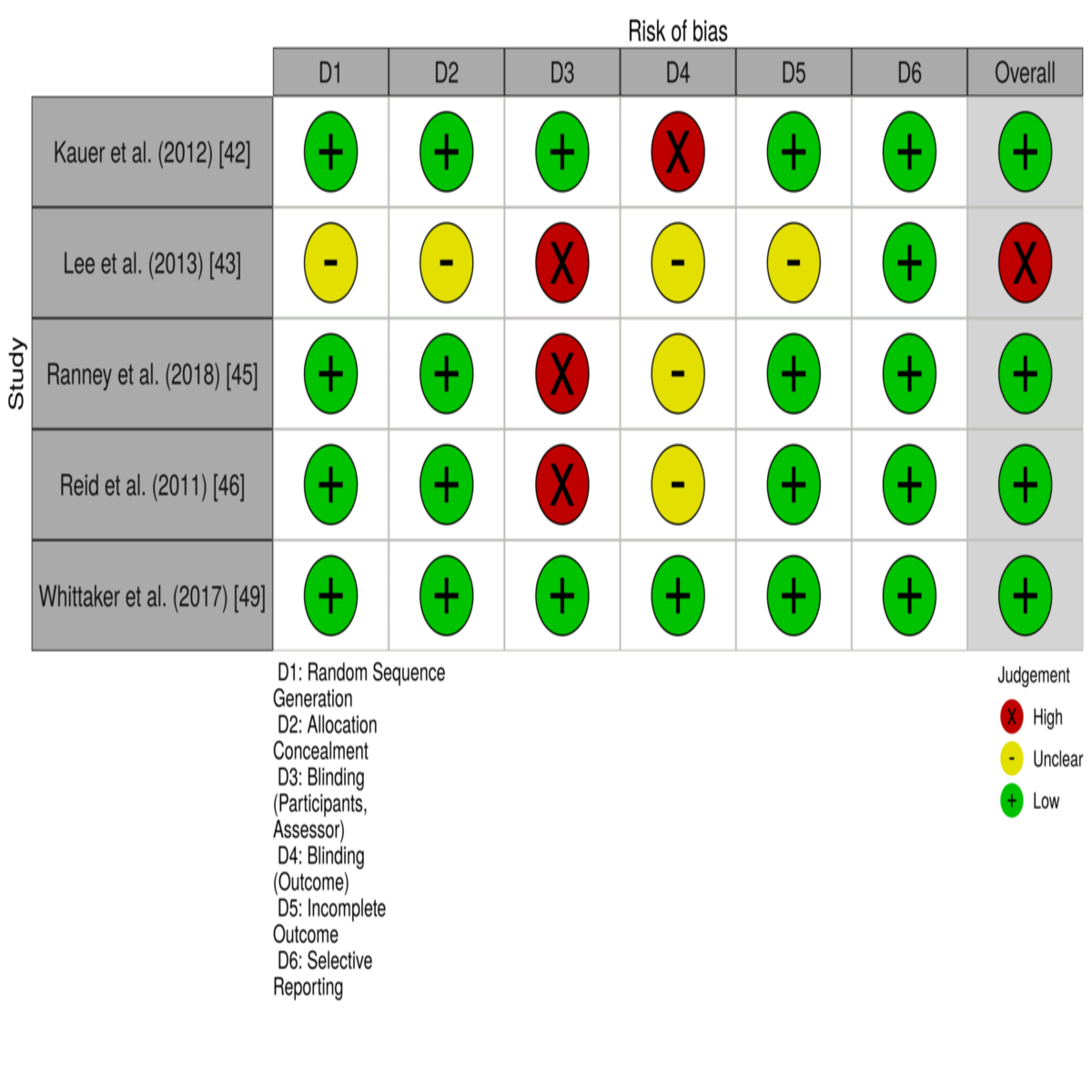

Supplement: Multimedia Appendix 2 [file jmir_v23i1e16490_app2.png]

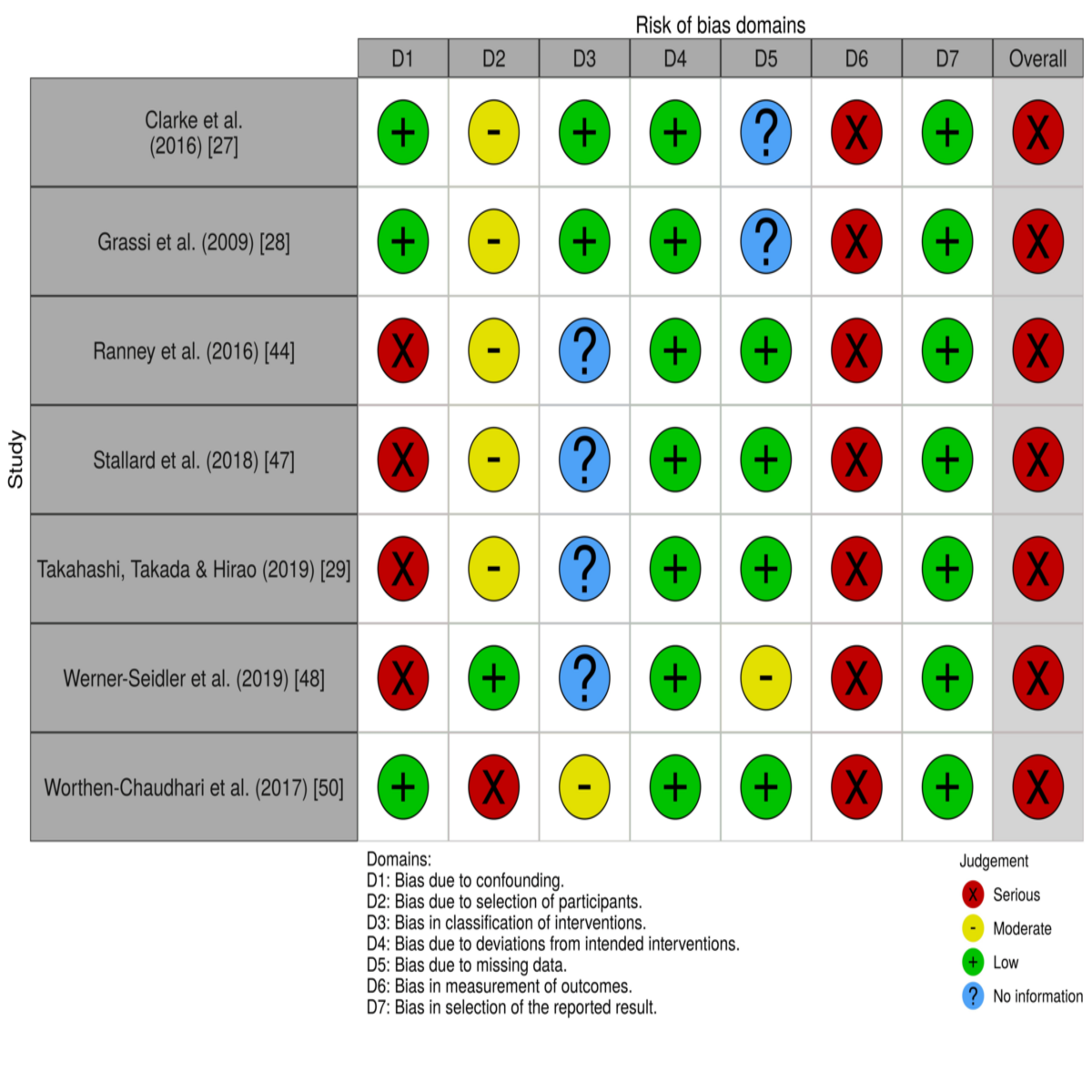

Supplement: Multimedia Appendix 3 [file jmir_v23i1e16490_app3.png]
